# Late Surgeon-General Hammond

**Published:** 1864-09

**Authors:** A. Lincoln


### EDITORIAL DEPARTMENT.

### LATE SURGEON-GENERAL HAMMOND.

In our last issue we published the charges preferred against the late Surgeon-General, which it appears by the finding of the court were fully sustained. It is painful to be obliged to confess a member of our profession guilty of such crimes as render him unworthy of holding any "office of honor, profit or trust under the government of the United States." The trial lasted for nearly four months; the court was composed of nine general officers; the trial, it is said, was the most patient and thorough, and after prolonged investigation terminated with the verdict of guilty of the charges preferred. Dr. Hammond has invited the public to withhold its judgment until he has made his defence, so perhaps we are not at liberty to comment too freely upon his character or conduct. It appears by the charges, when they are divested of all superfluous expression, that the Surgeon-General ordered, or caused to be purchased, blankets at extravagant prices, considering quality; that they were unfit for military hospitals was not sustained. That he directed the purchase of medical supplies for the army, of inferior quality and extravagant price. That he wrote some things concerning the Medical purveyor in Philadelphia which were not strictly true, and was ungentlemanly enough to omit the General in writing of General Halleck. That a surgeon should be guilty of any such charges is truly humiliating, and we do not desire to make paliation or extension of the crime in the least degree. There appears no ground for supposing that the above charges were not fully sustained; the order of the President confirms this conclusion, and leaves no doubt that the evidence was conclusive.

We suppose we must grant all that is charged, and acknowledge that Dr. Hammond manifested a dishonest intention to favor some of his friends—to help them sell some poor blankets at an extravagant price. If he had not been surrounded by the temptations attendant upon office, and constantly exposed to the effects of example in official circles, he would probably never have committed any such indiscretion. We honestly believe that such conduct is much too near the general rule, and that a higher standard of honesty is unknown in such quarters, only in exceptional cases. In our estimation, the charge of buying blankets at great prices is an accusation which would be very likely to be urged by somebody who had blankets to sell, and would have been willing to have found a similar market. It is no paliation of crime that it is fashionable, and still this crime of buying army supplies at such prices that favorites may realize handsome profits, is a thing which custom well nigh sanctions; it is a part of the plan, which is generally understood, but when expressed and exposed, looks badly.

When men come to do things which are not strictly honest, it is very common for them to say and write what is not true. This is very unbecoming a gentleman, and especially an officer clothed with the most important and sacred duties—provision for the sick and wounded soldiers. If a court martial was ordered for the trial of most officers having similar duties—purchases of supplies for the army, or direction of the locations and and duties of other officers, it does not appear certain that Dr. Hammond's integrity and uprightness of action would suffer in comparison; when tried in the exact balance of truth and purity, all would be found wanting. That he transcended the line of his duty and favored his friends at the expense of the government is about what appears from the report of the trial.. If the official conduct of Dr. Hammond was so correct as to require four months to collect evidence of his having paid extravagant prices for army supplies, we are of opinion his official purity was unequalled.

Our cotemporaries speak of this matter, as of an unparalleled corruption and villainy, but we think possibly there are extenuating circumstances which will yet be made to appear; but if there are none, we are disposed to rank it with the crimes which are of so common occurrence as to demand general reformation as much as individual punishment.

"Disqualified from holding office!" If he has done a "nice thing for his friends," he will be the favorite candidate for the next election. "Honesty in party and politics is nowhere." and it will never be found to prevail, as a rule, until it comes to be expected and more rigidly required of those who occupy places of honor and trust. Of the present army of office-holders on the one hand, and the still greater army of office seekers on the other, "whoever is without sin, let him cast the first stone."

The following is the finding of the court-martial:

1. The Court, after due and mature deliberation upon the evidence adduced, do find the accused, Brigadier-General William A. Hammond, Surgeon-General United States Army, as follows:

CHARGE I.

Of the 1st *Specification*, "Guilty,"

Of the 2d *Specification*, "Guilty," and that the offence therein charged was committed on the 30th day of May, A. D. 1863, except as to the words, " and with intent to aid private persons resident in Philadelphia," and as to which words so excepted, " Not Guilty.

Of the 3d *Specification*, " Guilty."

Of the 4th *Specification*, " Guilty," except as to the words, " and which blankets so ordered were unfit for hospital use," and as to which words so excepted, " Not Guilty."

Of the 5th *Specification*, " Guilty."

Of the 6th *Specification*, " Guilty."

Of the 7th *Specification*, " Guilty," except as to the words, " corruptly and," " and which beef so ordered was of inferior quality, unfit for hospital use, unsuitable and unwholesome for the sick and wounded in hospitals, and not demanded by the exigencies of the public service," and of the words so excepted, " Not Guilty."

Of the 8th *Specification*, "Not Guilty."

Of the 1st CHARGE, " Guilty."

CHARGE II.

Of the 1st *Specification*, " Guilty."

Of the 2d CHARGE, " Guilty."

CHARGE III.

Of the 1st *Specification*, " Guilty," except as to the words " and corruptly," " and cause," and as to which words so excepted, " Not Guilty."

Of the 2d *Specification*, " Guilty."

Of the 3d CHARGE, " Guilty."

SENTENCE.

And the Court do therefore sentence him, Brigadier-General William A. Hammond, Surgeon-General United States Army, " *That he shall be dismissed the service, and be forever disqualified from holding any office of honor, profit or trust, under the Government of the United States.*"

II..In compliance with the 65th Art. of the Rules and Articles of War, the whole proceedings of the General Court-Martial in the foregoing case have been transmitted to the Secretary of War, and by him laid before the President of the United Rtates.

The following are the orders of the President of the United States:

The record, proceedings, findings, and sentence of the Court in the foregoing case are approved: and it is ordered that Brigadier-General William a Hammond, Surgeon-General of the United States army, be dismissed the service, and be forever disqualified from holding any office of honor, profit, or trust, under the Government of the United States.

*August* 18, 1864. A. LINCOLN.

